# *Streptococcus pneumoniae* Enhances Human Respiratory Syncytial Virus Infection *In Vitro* and *In Vivo*

**DOI:** 10.1371/journal.pone.0127098

**Published:** 2015-05-13

**Authors:** D. Tien Nguyen, Rogier Louwen, Karin Elberse, Geert van Amerongen, Selma Yüksel, Ad Luijendijk, Albert D. M. E. Osterhaus, W. Paul Duprex, Rik L. de Swart

**Affiliations:** 1 Department of Viroscience, Erasmus MC, Rotterdam, The Netherlands; 2 Department of Medical Microbiology and Infectious Diseases, Erasmus MC, Rotterdam, The Netherlands; 3 National Institute of Public Health and the Environment, Bilthoven, The Netherlands; 4 Department of Microbiology, Boston University School of Medicine, Boston, MA, United States of America; Instituto Butantan, BRAZIL

## Abstract

Human respiratory syncytial virus (HRSV) and *Streptococcus pneumoniae* are important causative agents of respiratory tract infections. Both pathogens are associated with seasonal disease outbreaks in the pediatric population, and can often be detected simultaneously in infants hospitalized with bronchiolitis or pneumonia. It has been described that respiratory virus infections may predispose for bacterial superinfections, resulting in severe disease. However, studies on the influence of bacterial colonization of the upper respiratory tract on the pathogenesis of subsequent respiratory virus infections are scarce. Here, we have investigated whether pneumococcal colonization enhances subsequent HRSV infection. We used a newly generated recombinant subgroup B HRSV strain that expresses enhanced green fluorescent protein and pneumococcal isolates obtained from healthy children in disease-relevant *in vitro* and *in vivo* model systems. Three pneumococcal strains specifically enhanced *in vitro* HRSV infection of primary well-differentiated normal human bronchial epithelial cells grown at air-liquid interface, whereas two other strains did not. Since previous studies reported that bacterial neuraminidase enhanced HRSV infection *in vitro*, we measured pneumococcal neuraminidase activity in these cultures but found no correlation with the observed infection enhancement in our model. Subsequently, a selection of pneumococcal strains was used to induce nasal colonization of cotton rats, the best available small animal model for HRSV. Intranasal HRSV infection three days later resulted in strain-specific enhancement of HRSV replication *in vivo*. One *S*. *pneumoniae* strain enhanced HRSV both *in vitro* and *in vivo*, and was also associated with enhanced syncytium formation *in vivo*. However, neither pneumococci nor HRSV were found to spread from the upper to the lower respiratory tract, and neither pathogen was transmitted to naive cage mates by direct contact. These results demonstrate that pneumococcal colonization can enhance subsequent HRSV infection, and provide tools for additional mechanistic and intervention studies.

## Introduction

Respiratory tract infections cause a significant global burden of morbidity and mortality in all age groups. In 2011, the World Health Organization estimated that 1.3 million global deaths were due to acute respiratory infections, of which the majority occurred in developing countries in children under 5 years of age [1]. Of all respiratory pathogens *S*. *pneumoniae* or pneumococcus is the leading cause of death, accounting for more than 30% of the cases [1]. Many healthy children and adults carry pneumococci in their upper respiratory tract (URT) and *S*. *pneumoniae* colonizes the nasopharynx of up to 45% of children under 3 years of age and up to 20% of adults [2]. Colonization is a prerequisite for subsequent spread, which may result in respiratory tract infection (sinusitis, otitis media, pneumonia), meningitis or sepsis [3]. More than 90 *S*. *pneumoniae* serotypes have been identified, and pneumococcal vaccines have been developed containing antigens of up to 23 of the most important serotypes associated with human disease [4].

Human respiratory syncytial virus (HRSV) is a leading viral cause of respiratory tract infection in young infants [5]. HRSV is a member of the family *Paramyxoviridae*, a group of enveloped viruses with a single stranded RNA genome of negative polarity [6]. Despite the huge global burden of HRSV disease, no licensed vaccines are available [7]. Monoclonal antibody treatment (Palivizumab) is efficacious in high-risk infants [8], but the cost-effectiveness of this approach is under debate [9].

*S*. *pneumoniae* and HRSV occupy the same niche: pneumococcus colonizes the nasopharynx, where HRSV infects ciliated epithelial cells. In addition, peak prevalence of disease burden for both occurs in winter [10, 11], transmission occurs via direct contact and therefore crowding (e.g. in day care centers) contributes to spread of both pneumococci and HRSV [12–14]. Furthermore, risk factors for development of severe disease are highly similar for both pathogens, including premature birth, congenital heart disease, chronic lung disease and high age [13].

Several studies have described interactions between *S*. *pneumoniae* and HRSV [15–17]. Following the discovery of HRSV in 1956, it was described that pneumococci could be cultured from an HRSV-infected infant [18]. *In vitro* experiments showed that *S*. *pneumoniae* adheres to HRSV-infected human epithelial cells [19], and conversely, HRSV binds directly to pneumococci [20, 21]. In addition, HRSV infected mice showed significantly higher levels of pneumococcal bacteremia than controls [20]. Collectively these data suggest that HRSV infection can predispose individuals to bacterial superinfection, potentially leading to increased morbidity and mortality.

It has also been suggested that bacterial colonization may enhance respiratory virus infections. A potential mechanism was shown previously by synthetic bacterial lipopeptides-mediated enhancement of HRSV infection [22]. Pre-incubation of well-differentiated normal human bronchial epithelial (wd-NHBE) cells with *S*. *pneumoniae* enhanced human metapneumovirus (HMPV) infection [23]. In addition, frequent nasopharyngeal carriage of pneumococci in children under 2 years of age was associated with increased seroconversion rates to HMPV [23]. Moreover, in a clinical trial pneumococcal vaccination reduced the incidence of hospitalization for pneumonia associated with HRSV by 32% [24]. These observations indicate that pneumococcus and HRSV potentially have bidirectional interactions.

The aim of the current study was to assess the influence of pneumococcal colonization on subsequent HRSV infection using *in vitro* and *in vivo* models. We used wd-NHBE cells for *in vitro* studies and cotton rats (*Sigmodon hispidus*) for *in vivo* studies, as the most susceptible and disease-relevant HRSV model systems currently available. *S*. *pneumoniae* isolates had previously been obtained from nasopharyngeal brushes of healthy infants. One additional *S*. *pneumoniae* strain was obtained from ATCC. For HRSV infections we used a recently generated recombinant (r) wild-type subgroup B HRSV strain expressing enhanced fluorescent protein (EGFP; rHRSV^B05^EGFP(5)) [25]. We demonstrate that specific strains of *S*. *pneumoniae* can significantly and reproducibly enhance HRSV infection *in vitro* and/or *in vivo*.

## Materials and Methods

### Ethics statement

Animal experiments were conducted in strict accordance with European guidelines (EU directive on animal testing 86/609/EEC) and Dutch legislation (Experiments on Animals Act, 1997). The protocols were approved by the independent animal experimentation ethical review committee DCC in Driebergen, The Netherlands (approval number EMC2974). Animal welfare was monitored daily, and animal handling was performed under light anesthesia using isoflurane.

### *S*. *pneumoniae*, HRSV and cell culture conditions

Four low passage strains of *S*. *pneumoniae* were obtained from nasopharyngeal swabs of children under 2 years. Strain 19F was purchased from ATCC (cat. no. 49619). Bacteria were typed by capsular sequence typing [26, 27]. All strains were penicillin-sensitive, belonged to different serotypes, and will be referred to by the number of their serotype in this manuscript. Live bacterial numbers were determined by optical density at 600 nm and colony forming unit (CFU) counts obtained from serial dilutions. One day prior to each experiment bacteria were cultured twice at 35°C (5% CO_2_ [v/v]) to log phase on trypticase soy agar blood plates with 5% [v/v] sheep blood (TSA plates, BD). The following day, 12 hours after growth on the second plate bacteria were harvested in 15 ml tubes and OD_600_ values were determined. Bacterial suspensions were washed three times with PBS. Low passage (6 and 7) rHRSV^B05^EGFP(5) had been generated previously [25]. Virus was grown in HEp-2 cells, and purified by a two-step sucrose gradient centrifugation. The final stock contained approximately 25% (w/v) sucrose, and had a titer of 1.3x10^7^ TCID_50_/ml.

Mycoplasma-free HEp-2 cells (ATCC CCL-23) were grown in DMEM (Lonza) with 10% (v/v) fetal bovine serum (Sigma-Aldrich). Well-differentiated human normal bronchial epithelial (wd-NHBE, Lonza) cells were grown on air-liquid-interface in filters with 0.4 μm pore size (Corning) as described previously [23]. Importantly, cells were grown in antibiotics-free medium until after pneumococcus and/or virus inoculation. wd-NHBE cells were used 21 days after ALI, at which stage beating cilia and mucus production were clearly detectable.

### Animals

Four- to five-week-old female cotton rats (*Sigmodon hispidus*) were obtained from a specific pathogen-free breeding colony (Harlan). Two or three animals were housed in individual ventilated cages (IVC) supplemented with a tin can as hiding place (height 107 mm, diameter 72 mm) and paper tissues as cage enrichment and received food and water *ad libitum*. Experiments started after an acclimatization period of 7–10 days.

### wd-NHBE cells: co-infection and neuraminidase assays

Bacterial suspensions were prepared as described above. Ratios of bacteria to cells and virus to cells were 30:1 and 0.4:1, respectively, based on an estimated number of 25,000 cells on the apical surface. The mix (50 μl) was left onto the apical surface for 2 hours at 37°C (5% CO_2_ [v/v]), followed by aspiration of the supernatant. Four hours later the apical surface was washed three time with Dulbecco’s PBS at 37°C (100 μl) and antibiotics (penicillin (100 U/ml) and streptomycin [100 μg/ml]) were added to the basolateral compartment. Two d.p.i. automated whole well scans were made, followed by semi-automated enumeration of EGFP^+^ cells (DotCount, MIT, Boston).

To study the effect of neuraminidase the apical surface of wd-NHBE cells were treated with neuraminidase from *Vibrio cholerae* (Sigma-Aldrich #N7885) at two different concentrations (30 or 100 mU/ml) for one hour at 37°C. Cells were infected with rHRSV^B05^EGFP(5) (ratio 0.4:1) after washing the apical surface twice with DPBS. After another four hours wd-NHBE cells were washed three time and EGFP^+^ cells were enumerated 2 d.p.i.

Apical inocula of the wd-NHBE coinfection experiments were used to quantify pneumococcal neuraminidase activity using the NA-*Star* Influenza Neuraminidase Inhibitor Resistance Detection Kit (Applied Biosystems) according to manufacturer’s protocol. *Vibrio cholerae* neuraminidase was used as positive control and reference. Briefly, inocula were diluted 1:5 with NA-*Star* Buffer and incubated for 20 min with NA-*Star* chemiluminescent substrate. Assay plates were placed in a luminometer equipped with an on-board injector (Tecan Infinite M200). Light signal intensities were measured after injection of accelerator solution.

### Infection of colonized animals and *in vivo* transmission study

Animals were housed in groups of 2 or 3 animals per IVC. After an acclimatization period of one week, pneumococcal carriage was induced by intra-nasal (i.n.) inoculation of 5x10^5^ CFU in of PBS (10 μl). Three days later animals were inoculated i.n. with 1x10^4^ TCID_50_ (10 μl) and at day eight animals were euthanized by exsanguation. All procedures were performed under 4% (v/v) isoflurane anesthesia. Post-mortem nasopharyngeal lavages and lungs were collected for isolation of pneumococci and HRSV. Post-mortem nasopharyngeal lavages were obtained by flushing with virus transport medium (1 ml) without antibiotics from the proximal trachea towards the nasal cavity. Right lungs were homogenized with a M-tube (GentleMACS, RNA program 0.10.2) in virus transport medium (2 ml) without antibiotics [28] and centrifuged at 400 × g for 10 min. For determination of bacterial titers *S*. *pneumoniae* selective blood agar plates were custom made (trypticase soy agar blood plates with 5% (v/v) sheep blood and 5 μg/ml gentamycin) [29]. Plates were incubated at 35°C (5% [v/v] CO_2_) and CFU were counted manually the next day. Virus isolation from post-mortem nasopharyngeal lavages was performed as previously described [30, 31]. The lower limit of detection of bacteria was 10 CFU/ml and for HRSV detections in nasopharyngeal lavages or lung homogenates were 30 or 10 TCID_50_/ml, respectively. Nasal septum, conchae, one agarose-inflated lung were collected in 2% (w/v) paraformaldehyde for real-time monitoring of EGFP^+^ cells [28, 32].

### Microscopic detection of fluorescence

Microscopically, EGFP^+^ cells were identified by using an inverted fluorescence microscope (Zeiss Axiovert 25) or a confocal laser scanning microscope (Zeiss inverted AxioObserver Z1 equipped with LSM 700 scanning module). Nasal septum used for indirect immunofluorescence staining was transferred to PBS, permeabilized with 0.1% (v/v) Triton-X100 for 30 min and subsequently stained. TO-PRO-3 (Invitrogen) was used to counterstain nuclei (red). All images were generated using Zen 2010 software (Zeiss).

### Statistical analysis

All *in vitro* experiments were performed at least three times using triplicate or quadruple measurements. Statistical analyses were performed with SPSS version 20.0. Mann–Whitney U-tests were used to compare differences. A two-sided *p*-value ≤ 0.05 was considered statistically significant.

## Results

### *Specific S*. *pneumoniae* strains enhance HRSV infection *in vitro*

**Fig 1:**
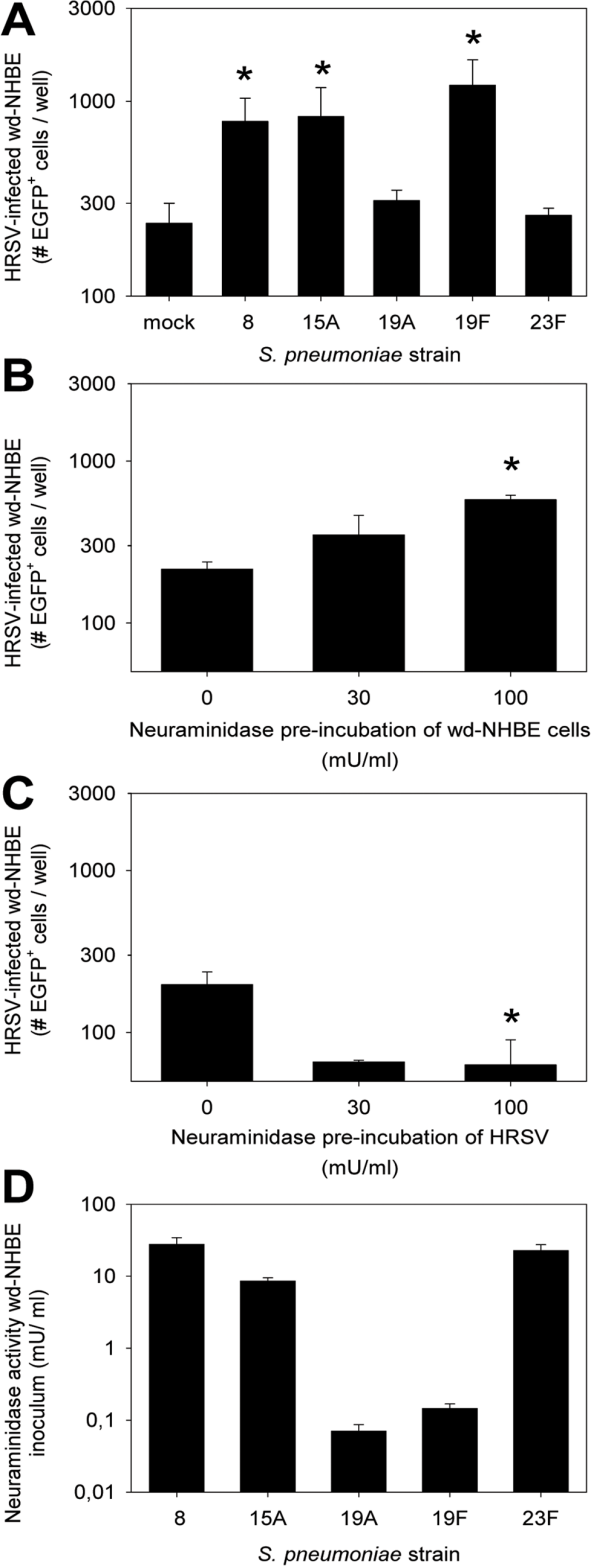
Enhancement of HRSV infection mediated by specific *S*. *pneumoniae* strains in wd-NHBE cells. (A) wd-NHBE cells were inoculated with HRSV and one *S*. *pneumoniae* strain with mock control. Strain 8, 15A and 19F enhanced HRSV infection as evidenced by quantification of EGFP^+^ cells 2 d.p.i. Data are represented as mean ± SD. * *p* ≤ 0.05; 2-tailed Mann-Whitney *U* test. (B) wd-NHBE cells were treated with neuraminidase from *Vibrio cholerae*, followed by HRSV infection after washing. Two dpi the numbers of HRSV-infected cells were enumerated. Data are presented as means ± SD. * *p* ≤ 0.05; 2-tailed Mann-Whitney *U* test. (C) HRSV was treated with neuraminidase from *Vibrio cholerae* before inoculation of wd-NHBE cells. Treated virus was incubated for 1 hour, followed by washing. HRSV-infected cells were enumerated 2 dpi. Data are presented as means ± SD. * *p* ≤ 0.05; 2-tailed Mann-Whitney *U* test. (D) Apical inocula of the wd-NHBE cell co-infection experiment shown in Fig 1A were tested for neuraminidase activity using the NA-*Star* Influenza Neuraminidase Inhibitor Resistance Detection Kit (Applied Biosystems). Numbers correspond to *Vibrio cholerae* μU neuraminidase activity. Data are presented as means ± SD.

We used wd-NHBE cells grown on air-liquid interface expressing beating cilia and goblet cells as the best available *in vitro* model for HRSV infection. Also, we used rHRSV^B05^EGFP(5), which is the first HRSV strain displaying the full phenotype and genetic background of currently circulating wild-type viruses [25]. Along these primary cells and recombinant virus low passage pneumococci were used to mimic the complex interplay between pneumococcus and HRSV. Initially the apical surface of wd-NHBE was incubated with *S*. *pneumoniae*, but epithelia cultured without antibiotics rapidly deteriorated and died. Therefore, we used an alternative approach, in which wd-NHBE cells were incubated with a mixture of streptococci and HRSV in the absence of antibiotics. Virus and bacteria were removed after 2 hours, and 4 hours later the apical surface was washed with PBS supplemented with antibiotics and the basolateral medium was replaced by medium with antibiotics ensuring survival of the cultures. Co-administration of HRSV with three pneumococcus strains (belonging to serotypes 8, 15A and 19F) resulted in significantly increased numbers of HRSV-infected cells (*p*<0.05), but two other strains (belonging to serotypes 19A and 23F) did not modulate HRSV infection (Fig 1A).

### A potential role of bacterial neuraminidase?

It was previously described that neuraminidase treatment of HRSV-infected cells enhanced cell-cell fusion and infection in an epithelial cell line [33]. Thus, we hypothesized that neuraminidase could also influence fusion between the viral and host cell membranes. Treatment of wd-NHBE cells with commercially available bacterial neuraminidase of *Vibrio cholerae* resulted in a dose-dependent enhancement of HRSV infection (Fig 1B). However, in contrast to the observations published by Barretto *et al*., we only observed enhancement of infection when polarized cells were treated, and inhibition when the virus was pre-treated with neuraminidase (Fig 1C). To assess the potential role of bacterial neuraminidases in the observed enhancement of HRSV, we measured the neuraminidase activity of the virus / bacterium mixture after removal from the apical surface of the wd-NHBE cells shown in Fig 1A. However, neuraminidase activity did not correlate with the observed pattern of enhancement of HRSV infection (Fig 1D).

### *Streptococcus pneumoniae* colonization can enhance HRSV infection *in vivo*

**Fig 2:**
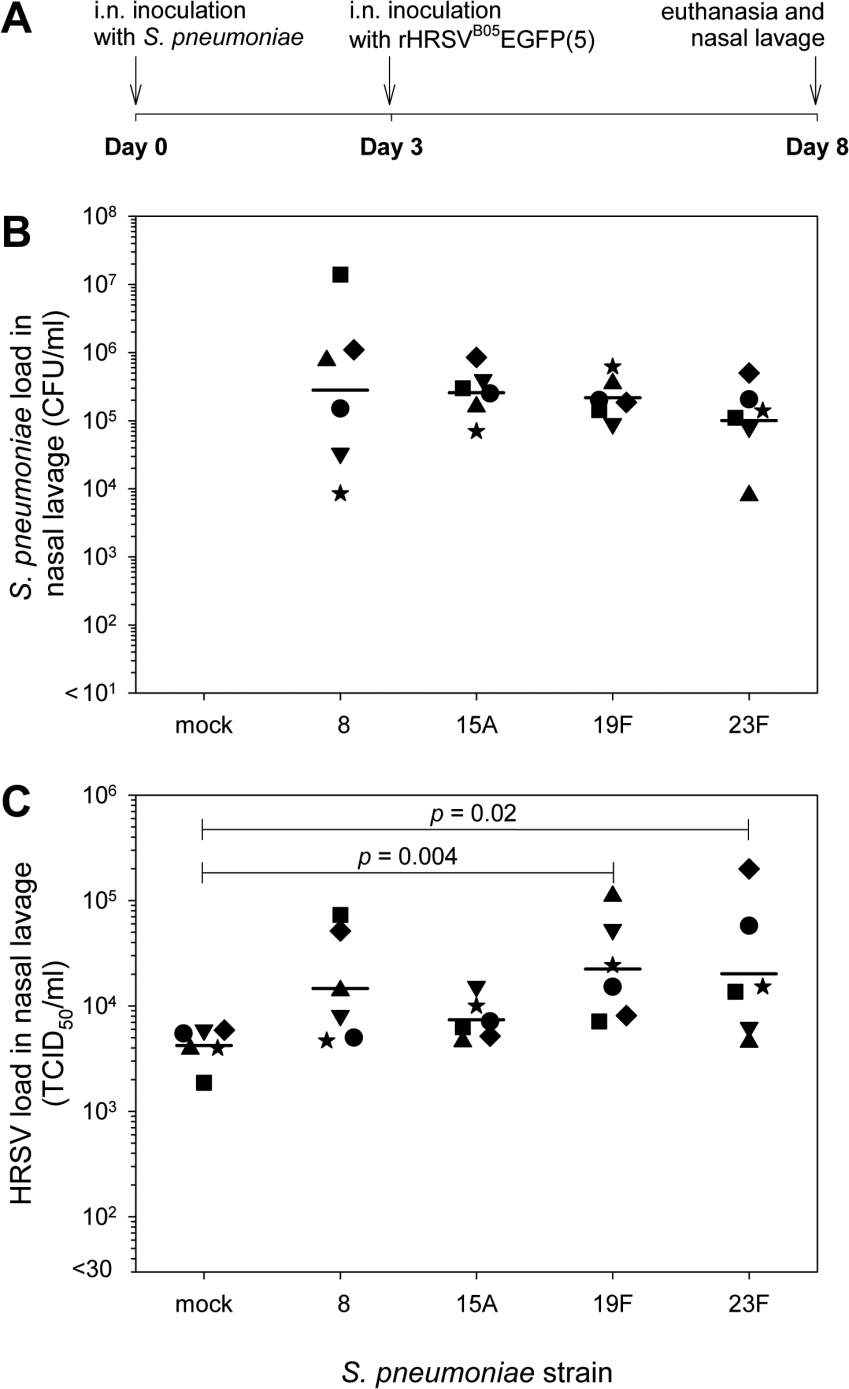
Bacterial and virus titers of HRSV infected *S*. *pneumoniae* colonized cotton rats. (A) Experimental design. Pneumococcal carriage was induced by i.n. inoculation of cotton rats with 5x10^5^ CFU *S*. *pneumoniae* in 10 μl at day 0. Three days later animals were infected with 1x10^4^ TCID_50_ rHRSV^B05^EGFP(5) in PBS (10 μl). At day 8 animals were euthanized. (B) Bacterial titers eight days after induction of pneumococcal carriage. The geometric mean titer was about 2x10^5^ CFU/ml for the different strains. Data symbols represent individual animals, bars represent geometric mean titers (GMT) per group. * *p* ≤ 0.05; 2-tailed Mann-Whitney *U* test. (C) Virus titers five days after HRSV infection. Significantly higher virus load were detected in groups with nasal carriage of *S*. *pneumoniae* strain 19F and 23F compared to mock-treated. * *p* ≤ 0.05; 2-tailed Mann-Whitney *U* test.

In order to extrapolate our *in vitro* results a new *in vivo* pneumococcal colonization & HRSV infection model was developed. We established this model using four *S*. *pneumoniae* strains and the recombinant HRSV strain expressing EGFP (Fig 2A). Animals (n = 6/group) were intra-nasally inoculated with 5x10^5^ CFU of pneumococci or with PBS as mock control. An inoculum volume of 10 μl was used to prevent primary inoculation of the lower respiratory tract (LRT) [34]. Animals were infected 3 days post-colonization with 1x10^4^ 50% tissue culture infectious doses (TCID_50_) of rHRSV^B05^EGFP(5) in PBS (10 μl). No samples were collected until euthanasia at day 8 (i.e. day 5 after HRSV infection) to prevent any mechanical/physical interference with bacterial colonization or viral infection and spread. Nasopharyngeal lavages and lung homogenates were collected post-mortem for isolation of pneumococci and HRSV. Nasal septum, conchae and agarose-inflated lungs were collected for immediate monitoring of EGFP^+^ cells [28, 32].

Bacteria were re-isolated from nasopharyngeal lavage from all animals intra-nasally inoculated with *S*. *pneumoniae* (Fig 2B). The geometric mean titers were about 2x10^5^ CFU/ml, with the highest variation for strain 8. Bacteria could not be cultured from homogenized lungs of any of the animals. At the same time point, rHRSV^B05^EGFP(5) was isolated from nasopharyngeal lavages of all animals (Fig 2C). The geometric mean titers were 4.2x10^3^, 1.5x10^4^, 7.4x10^3^, 2.2x10^4^ and 2.0x10^4^ TCID_50_/ml for the groups receiving mock or strains 8, 15A, 19F and 23F, respectively. Significantly higher virus loads were detected in groups inoculated with strains 19F and 23F (*p*<0.05) compared to the mock infection. For the other two strains there appeared to be a trend towards higher HRSV loads, but these did not reach statistical significance. Furthermore, the variation in virus loads within the colonized groups appeared to be higher.

**Fig 3:**
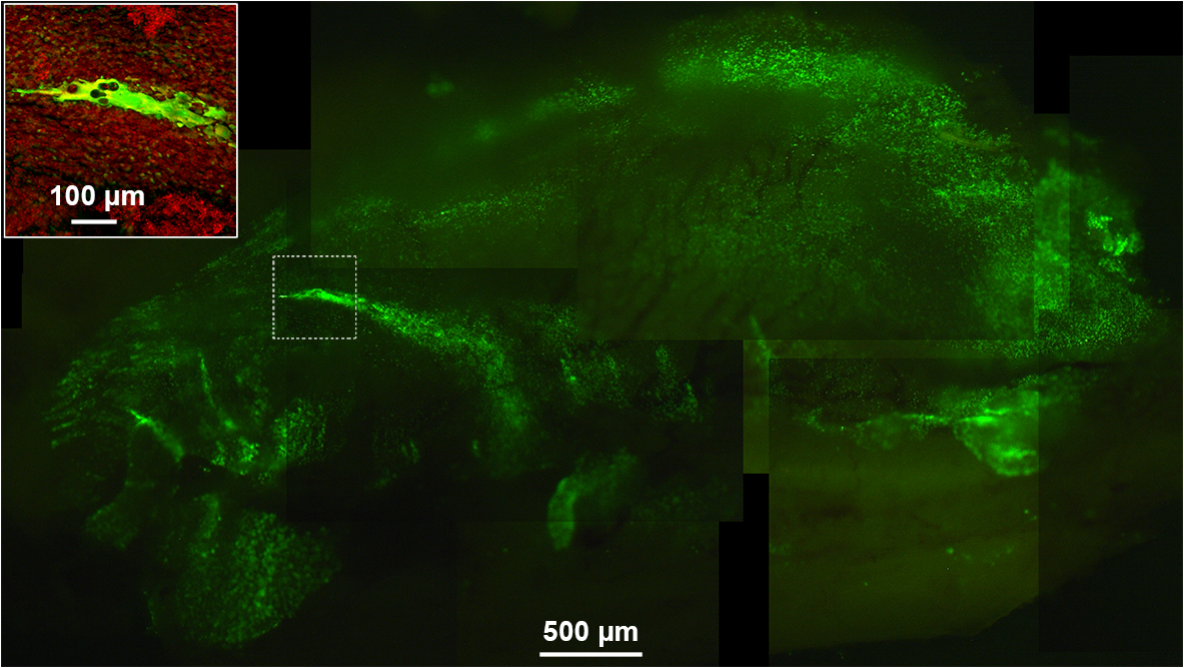
HRSV infection in nasal septum of a *S*. *pneumoniae* colonized cotton rat. Composite microscopic UV images of the complete nasal septum of a cotton rat 5 d.p.i. showing a representative syncytium in the middle (scale bar represents 500 μm). The inset is a confocal laser scanning image of the same nasal septum with nuclei stained with TO-PRO3 (red; scale bar represents 100 μm).

Upon necropsy, EGFP^+^ cells were detected in the nasal septum and conchae of all animals. No differences in numbers of EGFP^+^ cells were observed between the different groups, although absolute enumeration of these cells was impossible. Syncytia were observed in the nasal septum and conchae in 5 out of 6 animals of the group inoculated with *S*. *pneumoniae* strain 19F as compared to 1 out of 6 animals of the mock group (*p*<0.05, Fig 3). In the groups that had received *S*. *pneumoniae* strains 8, 15A or 23F these were seen in 1, 1 and 3 animals, respectively (not significantly different from the mock control). A small number of EGFP^+^ cells was found in the lungs in two animals that had received *S*. *pneumoniae* 19F or 23F (data not shown).

### Cotton rat transmission study

**Fig 4:**
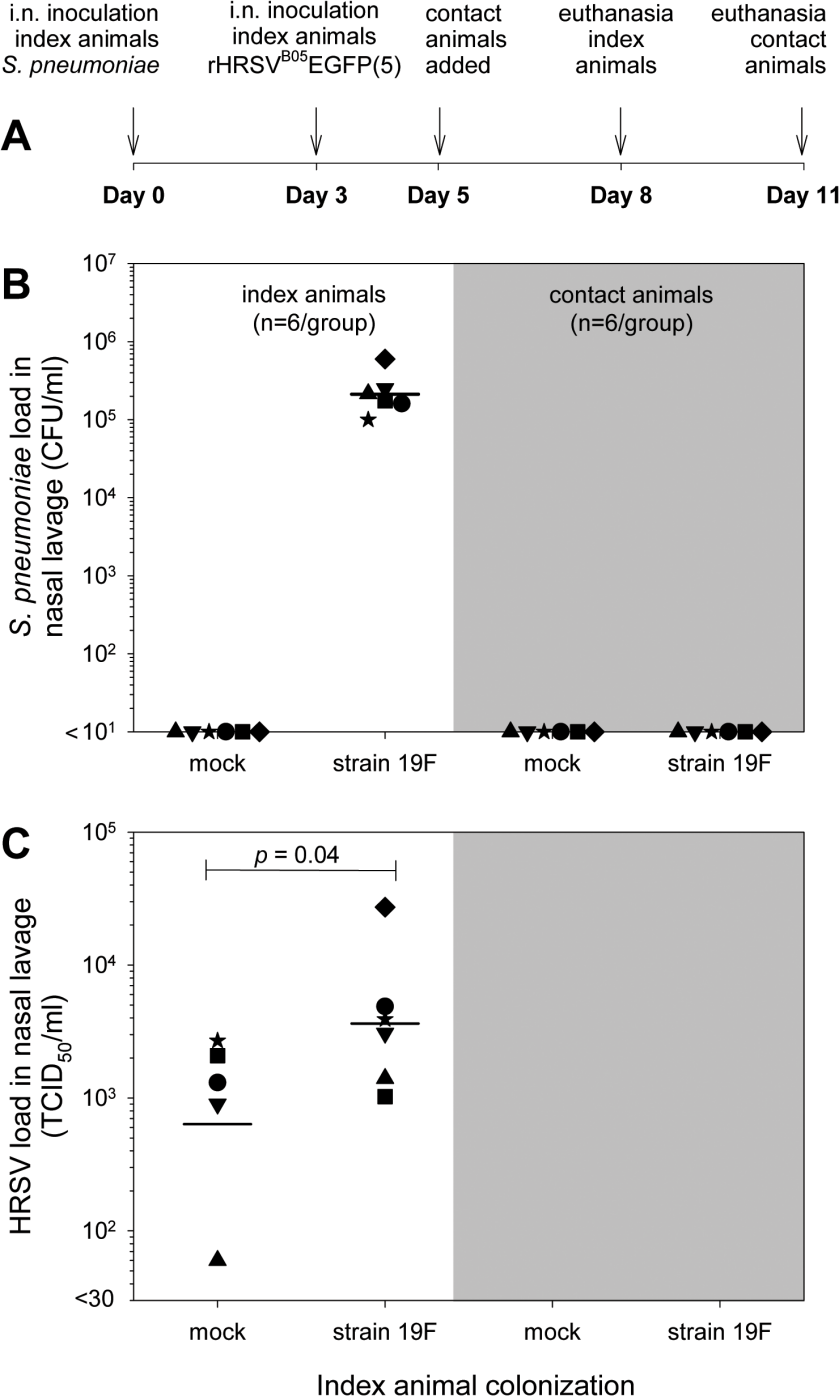
*In vivo* transmission study HRSV infection in *S*. *pneumoniae* colonized cotton rats. (A) Schematic representation of the time course of the experiment. Animals were initially housed in pairs. One of each pair was pneumococcus- or mock-colonized, and infected with HRSV three days later. Another two days later the matching naive contact animals were added to their cage, reuniting the original pairs. Index and contact animals were euthanized at day 8 or 11, respectively. (B) Bacterial titer of index and contact animals. The geometric mean titer (GMT) was about 2x10^5^ CFU/ml for the index group, but no bacteria were isolated in the contact animals. Each data symbol represents one animal, bars represent GMT. (C) Virus titers of index and contact animals. Significantly higher virus loads were detected in the index group colonized with strain 19F, as compared to mock-colonized. In both contact groups no virus could be isolated out of the nose and lungs. * *p* ≤ 0.05; 2-tailed Mann-Whitney *U* test.

*S*. *pneumoniae* strain 19F had enhanced HRSV infection both *in vitro* and *in vivo*. We hypothesized that higher virus loads could facilitate transmission of virus or bacteria to naive cage mates by direct contact. To test this hypothesis, and confirm the pneumococcal enhancement of HRSV infection *in vivo*, we designed a transmission experiment in cotton rats (Fig 4A). Two groups of index animals (n = 6) were mock-treated or intra-nasally inoculated with *S*. *pneumoniae* strain 19F, with each animal being housed solitarily. Three days later all index animals were infected intra-nasally with rHRSV^B05^EGFP(5). At day 5 naive contact animals were added to each cage permitting direct physical contact between the index and contact animals for 72 hours, including the anticipated peak of HRSV replication in the index animals. Index and contact animals were euthanized at days 8 and 11, respectively (Fig 4A). Bacterial and viral loads were determined in nasopharyngeal lavages and lung homogenates. In the colonized index group *S*. *pneumoniae* was cultured from the nasopharyngeal lavage of all animals, with a geometric mean titer of 2.1x10^5^ CFU/ml (Fig 4B). In addition, significantly higher virus loads were detected in the colonized index animals as compared to the mock control index animals (Fig 4C; *p*<0.05). On day 11 all contact animals were euthanized, and bacterial culture and HRSV re-isolation procedures were repeated. However, no *S*. *pneumoniae* or HRSV was detected in either the nose or the lungs of any of these animals (Fig 4C). Thus, *in vivo* enhancement of HRSV infection by nasal colonization of cotton rats with *S*. *pneumoniae* strain 19F proved to be reproducible, but we could not detect bacterial or viral transmission.

## Discussion

We have shown that specific *S*. *pneumoniae* strains enhance HRSV infections both *in vitro* and *in vivo*. In wd-NHBE cells simultaneously inoculated with HRSV and pneumococcus strain 8, 15A or 19F, but not strain 19A and 23F, significantly higher numbers of HRSV-infected epithelial cells. In cotton rats intra-nasally colonized with *S*. *pneumoniae* HRSV replicated to higher titers. However, in this case strains 19F and 23F resulted in enhancement of HRSV infection, whereas for strains 8 and 15A only a trend towards higher virus loads was detected. Strain 19F also promoted syncytium formation in the upper respiratory tract.

Co-infections with respiratory viruses and bacteria have been described, although their number is limited. Two prospective studies showed that 40% of children admitted to the pediatric intensive care unit with severe HRSV disease had a bacterial co-infection in the lower respiratory tract [16, 35, 36]. Similar observations have been described for influenza virus infections. During the influenza H1N1 pandemic in 1918–1919, an era in which no antibiotics were available, the majority of deaths was caused by *S*. *pneumoniae*, [37, 38]. In the most recent influenza virus pandemic starting in 2009 co-infection with *S*. *pneumoniae* was found to be associated with severe disease [39]. Diavatopoulos *et al*. found that influenza virus infection facilitated *S*. *pneumoniae* transmission and disease in suckling mice [40]. In addition to *S*. *pneumoniae*, *Haemophilus influenzae* and *Staphylococcus aureus* were also associated with influenza virus infection [41].

Studies on bacterial colonization modulating virus infections are scarce. Sajjan *et al* reported that *H*. *influenzae* can potentiate airway epithelial cell responses to rhinovirus by increasing ICAM-1 and TLR3 expression *in vitro* [42]. However, these authors used an eight-hour apical incubation period of wd-NHBE cells, which in our experience can result in a significant reduction in transepithelial electric resistance. Kuss *et al*. studied bacterial and viral interactions in the enteric tract and showed that the intestinal microbiota promoted poliovirus replication and systemic pathogenesis via binding to bacterial lipopolysaccharide [43]. Previously, we reported interactions between *S*. *pneumoniae* and HMPV infection *in vitro* and *in vivo* [23]. Madhi *et al*. showed in a large randomized clinical trial that pneumococcal conjugated vaccination reduced HRSV pneumonia in human immunodeficiency virus (HIV)-1-uninfected children [24]. Interestingly, bacteria can also protect from pneumovirus infections. Nasally administered immunobiotics (immunoregulatory probiotic lactic acid bacteria) differentially modulate respiratory antiviral immune responses, thereby inducing protection against pneumonia virus of mice or the closely related HRSV [44, 45].

Our *in vitro* co-cultures of *S*. *pneumoniae* and HRSV in wd-NHBE cells resulted in enhancement of HRSV infection mediated by *S*. *pneumoniae* strains belonging to serotypes 8, 15A and 19F, but not by strains belonging to serotypes 19A and 23F. However, since we tested only one strain of each of these serotypes, we cannot conclude that these differences are serotype-related, i.e. mediated by capsular structures. We considered a potential role of pneumococcal neuraminidases to explain the differences between these strains. Barretto *et al*. demonstrated that neuraminidase can enhance HRSV infection [33]. They found that treatment of HRSV with neuraminidases of different bacteria led to enhancement of infection. Neuraminidases enzymatically remove sialic acids, which could result in improved interaction between an HRSV transmembrane glycoprotein and its cellular receptor. We repeated these studies with rHRSV^B05^EGFP(5) in HEp-2 cells, a larynx carcinoma cell line, and confirmed the previously reported findings (data not shown). However, neuraminidase treatment of rHRSV^B05^EGFP(5) resulted in a dose-dependent inhibition of HRSV infection in wd-NHBE cells. In contrast, neuraminidase treatment of wd-NHBE cells resulted in a dose-dependent enhancement of HRSV infection. We speculate that this difference is primarily related to the presence of mucus in wd-NHBE cells. Neuraminidase treatment of the epithelial surface may result in disruption of the mucus layer [46, 47], while neuraminidase treatment of the virus may result in surface charge changes that promote HRSV interactions with mucus and inhibit HRSV binding to the target cells. Conclusive evidence for a potential role of neuraminidase in the infection enhancement could be obtained by testing pneumococcal strains with or without deletions of the neuraminidase genes (*NanA* and *NanB*) in our assay systems. However, we were unable to perform these experiments in the current study.

Pneumococcal carriage may have detrimental consequences as the bacterium can spread to other parts of the human body. Pneumococcal vaccination has been proven effective in preventing colonization [48]. Current vaccines include up to 23 of the most prevalent and disease-relevant strains [48]. In our study we have used strains 19F and 23F which are included in current vaccine formulations, whereas strains 8 and 15A are not. As global prevalence of serotypes included in the vaccine diminishes, other serotypes may fill the niche. This phenomenon is also referred to as serotype replacement [49–51].

In order to study the effect of pneumococcal carriage on HRSV infection we set up a new pneumococcal colonization & HRSV infection cotton rat model. To our knowledge this is the first study describing induction of pneumococcal carriage in cotton rats. *S*. *pneumoniae* are autolytic at the end of the log-phase, which is caused by the major enzyme *Lyt A* [52, 53]. Therefore, demonstration of high pneumococcal loads in nasopharyngeal lavages, but not in lung homogenates, 8 days after intra-nasal inoculation in 100% of the animals supports efficient pneumococcal colonization in the URT. Pneumococcal colonization appeared not to be detrimental to the animals, as no physical or behavioral signs of disease were observed. Body weights were minimally affected: all animals gained weight during the experiment but in the groups colonized with strains 8 and 15A there was a trend towards reduced gain in weights (data not shown). However, the differences were not significant and these two strains were not associated with significant enhancement of HRSV infection *in vivo*.

Interestingly, HRSV titers of mock-treated animals were quite homogeneous. In contrast, in some of the colonized animals with (enhancing) pneumococcal strains HRSV titers were almost 100-fold higher than those in the mock controls. These animals could potentially be considered as “superspreaders” [54, 55]. However, in this cotton rat model no transmission of either HRSV or *S*. *pneumoniae* could be detected, even when index and contact animals were housed together in the same cage. None of the animals displayed any clinical signs of HRSV upper respiratory tract infection such as rhinorrhea, sneezing, or coughing, reducing the chance of transmission to naive animals. We conclude that this model using adult cotton rats is not suitable for HRSV transmission studies.

In conclusion, using a new molecular clone of HRSV that mimics current wild-type strains in the genuine target cells (wd-NHBE) and a new pneumococcal colonization & HRSV infection animal model we have shown that *S*. *pneumoniae* modulates HRSV infections.
